# Recuperative Amino Acids Separation through Cellulose Derivative Membranes with Microporous Polypropylene Fiber Matrix

**DOI:** 10.3390/membranes11060429

**Published:** 2021-06-05

**Authors:** Aurelia Cristina Nechifor, Andreia Pîrțac, Paul Constantin Albu, Alexandra Raluca Grosu, Florina Dumitru, Ioana Alina Dimulescu (Nica), Ovidiu Oprea, Dumitru Pașcu, Gheorghe Nechifor, Simona Gabriela Bungău

**Affiliations:** 1 Analytical Chemistry and Environmental Engineering Department, University Politehnica of Bucharest, 1-7 Polizu St., 011061 Bucharest, Romania; aureliacristinanechifor@gmail.com (A.C.N.); andreia.pascu@yahoo.ro (A.P.); oanaalinadimulescu@yahoo.com (I.A.D.); dd.pascu@yahoo.com (D.P.); ghnechifor@gmail.com (G.N.); 2 IFIN Horia Hulubei, Radioisotopes and Radiation Metrology Department (DRMR), 30 Reactorului St., 023465 Măgurele, Romania; paulalbu@gmail.com; 3 Department of Inorganic Chemistry, Physical Chemistry and Electrochemistry, University Politehnica of Bucharest, 1-7 Polizu St., 011061 Bucharest, Romania; d_florina@yahoo.com (F.D.); ovidiu73@yahoo.com (O.O.); 4 Faculty of Medicine and Pharmacy, University of Oradea, Universităţii St., no.1, Bihor, 410087 Oradea, Romania; simonabungau@gmail.com

**Keywords:** amino acids, cellulose derivatives, polypropylene hollow fibers, impregnated membranes, amino acid separation, membrane processes

## Abstract

The separation, concentration and transport of the amino acids through membranes have been continuously developed due to the multitude of interest amino acids of interest and the sources from which they must be recovered. At the same time, the types of membranes used in the sepa-ration of the amino acids are the most diverse: liquids, ion exchangers, inorganic, polymeric or composites. This paper addresses the recuperative separation of three amino acids (alanine, phe-nylalanine, and methionine) using membranes from cellulosic derivatives in polypropylene ma-trix. The microfiltration membranes (polypropylene hollow fibers) were impregnated with solu-tions of some cellulosic derivatives: cellulose acetate, 2-hydroxyethyl-cellulose, methyl 2-hydroxyethyl-celluloseand sodium carboxymethyl-cellulose. The obtained membranes were characterized in terms of the separation performance of the amino acids considered (retention, flux, and selectivity) and from a morphological and structural point of view: scanning electron microscopy (SEM), high resolution SEM (HR-SEM), Fourier transform infrared spectroscopy (FT-IR), energy dispersive spectroscopy (EDS) and thermal gravimetric analyzer (TGA). The re-sults obtained show that phenylalanine has the highest fluxes through all four types of mem-branes, followed by methionine and alanine. Of the four kinds of membrane, the most suitable for recuperative separation of the considered amino acids are those based on cellulose acetate and methyl 2-hydroxyethyl-cellulose.

## 1. Introduction

**Figure 1:**
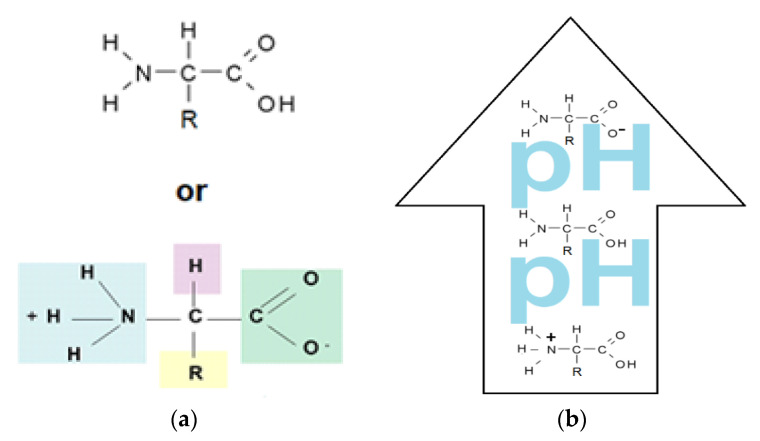
The chemical structure of amino acids (**a**) and the ionic forms depending on the pH of aqueous solution in which they are found (**b**).

Amino acids are substances whose biological role, when incorporated into protein molecules, neurotransmitters, or hormones and in precursors of other molecules, has led to a continuous development of fundamental and applied research [1,2]. The simultaneous presence in the molecule of amino acids, of a basic, amino group (-NH_2_) but also of an acid group, carboxyl (-COOH), close spatially (Figure 1a), gives these substances unique physical-chemical and biological properties [3,4]. Of course, one of the important aspects of this structure is given by the presence of electrical charges in amino acids [5,6,7], regardless of the pH of the aqueous solution in which they are found (Figure 1b). Simultaneously, the chemical properties of the amino and carboxyl groups make the amino acids the molecules of greatest interest in modifying, by grafting, the properties of various organic and inorganic materials [8,9,10,11].

All these characteristics, but also the multiple chemicals, biological and biomedical applications make it necessary to recover amino acids from any source, even when their concentration is low [12,13,14,15,16,17].

The properties of an amino acid will also be influenced by the R group, which can give the molecule specific interactions: hydrophobic, hydrophilic, or redox, depending on the atoms that make it up [18,19]. All these characteristics of amino acids allowed researchers to address the most varied techniques and methods of separation, among which some of those involving membranes are presented in Table 1 [20,21,22,23,24,25,26,27,28,29,30,31,32,33,34,35,36,37,38,39,40,41,42,43,44,45,46,47,48,49,50,51,52,53,54,55,56,57,58,59].

Sources of amino acid are among the most diverse, but mainly hydrolysates in the food industry have been addressed: milk, meat, fish, wine, or animal skin processing, agriculture, and biotechnology.

Each method or process of membrane separation, concentration, or purification of amino advantages and disadvantages and must be correlated with the processing system, so as to meet both selectivity and productivity requirements [60].

However, we must mention some of the specific performances of the membrane processes, which make them extremely attractive for the separation of compounds of biological interest such as the amino acids [51,52,53,54,55,56,57,58,59,60]:

- High selectivity;
- Productivity guided by membrane design;
- Accessible operating parameters;
- Raising the scale without multiplying the problems of chemical engineering;
- Reduced volume of installations;
- Access to the treatment of coarse and viscous dispersed systems;
- Minimizing of the consumption of chemical reagents and auxiliary materials;
- Reduced investments for the realization of the auxiliary systems;
- Complete automation;
- Possibility to operate in isolate or hard to reach locations;
- Favorable economic ratio, operating costs: product quality.

From the perspective of the application of the presented membrane processes (Table 1) for membrane separation of amino acids, they can be approached as follows:

- high flows and productivity [23,24,25,26,27],
- simplicity and robustness of the installations [28,29,30,31,32,33,34,35,36,37,38,39,40,41,42,43],
- or their selectivity [44,45,46,47].

The membranes can also be chosen according to selectivity and flow criteria [61,62,63], but in terms of biocompatibility, accessibility and cost the cellulose based membranes remain the most studied and applied [64,65].

This paper addresses the recuperative separation of three amino acids (alanine, phenylalanine, and methionine) from synthetic solutions, using membranes from cellulosic derivatives (cellulose acetate, 2-hydroxyethyl-cellulose, and methyl 2-hydroxyethyl-cellulose or sodium carboxymethyl-cellulose) in polypropylene hollow fiber matrix.

## 2. Materials and Methods

### 2.1. Materials

#### 2.1.1. Chemicals

The materials used in the present work were of analytical purity. They were purchased from Merck (Merck KGaA, Darmstadt, Germany): sodium hydroxide (NaOH) and hydrochloric acid solution (HCl, 35%).

The amino acids (alanine, phenylalanine and, methionine) and the cellulose derivatives (cellulose acetate (Product of USA), 2-hydroxyethyl-cellulose (product of USA), methyl 2-hydroxyethyl-cellulose (product of Belgium) and sodium carboxymethyl-cellulose (Product of USA)) were purchased from Aldrich Chemistry (Merck KGaA, Darmstadt, Germany).

The purified water, characterized by a conductivity of 18.2 µS/cm, was obtained with a RO Millipore system (MilliQR Direct 8 RO Water Purification System, Merck, Darmstadt, Germany).

#### 2.1.2. Membrane Support

**Figure 2:**
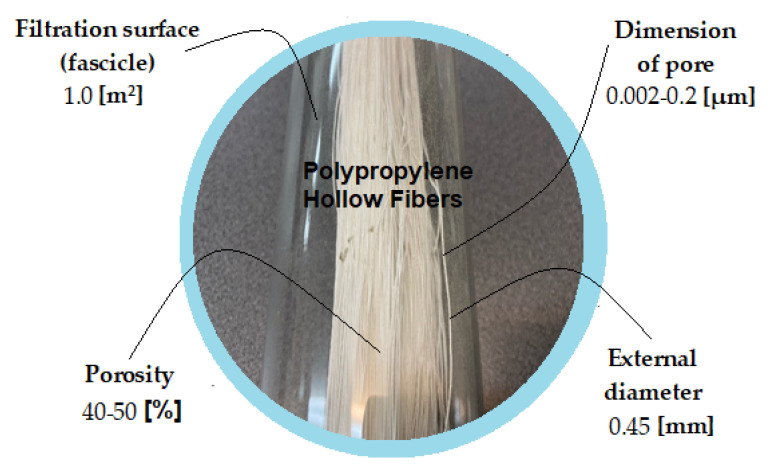
The characteristics of the hollow fibers polypropylene support membranes (PPM).

The hollow fibers polypropylene support membranes (PPM) were provided by GOST Ltd., Perugia, Italy. Their characteristics are presented in Figure 2 [66,67].

### 2.2. Impregnated Cellulose Derivatives Polypropylene Membrane Preparation (Cell-D-PPM)

#### 2.2.1. Obtaining the Cellulose Derivatives Solution

The cellulosic derivatives (cellulose acetate, 2-hydroxyethyl-cellulose, methyl 2-hydroxyethyl-cellulose or sodium carboxymethyl-cellulose), having the characteristics indicated in Table 2, were solubilized in a mass concentration of 2%, in a mixture of methylene chloride:methyl alcohol = 2:1.

For this, the glass vessel in which the mixture of solvents and the corresponding amount of polymer were introduced, was placed in an ultrasonic bath (Elmasonic S, Elma Schmidbauer GmbH, Singen, Germany) for four hours, observing the complete dispersion and obtaining the polymeric solution. The obtained solutions were filtered using a metal sieve with 40 µm × 40 µm meshes and then placed in closed containers for 24 h in order to remove bubbles.

#### 2.2.2. Obtaining Cellulose Derivatives Impregnated on Polypropylene Fibers Membranes (Cell-D-PPM)

**Figure 3:**
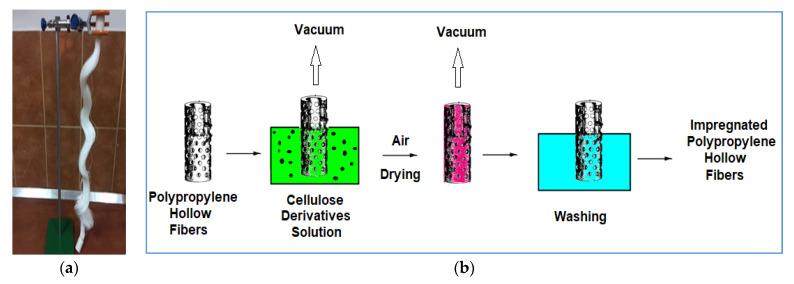
Schematic depiction of the impregnation procedure: (**a**) hollow polypropylene fiber bundles; and (**b**) scheme of the stages of the impregnation procedure.

For impregnation of support hollow fiber membranes, the polymer solution was placed in a glass cylindrical vessel with measured capacity and then the fiber bundle was immersed (Figure 3) with the heads out of the solution and coupled to a preliminary vacuum pump [68,69]. The fibers were coupled to the vacuum pump to remove the air and when the pressure difference increased, the pump switched-off automatically. The system was maintained in this state for 10 min, after which the impregnated fibers were removed above the polymer solution. After 30 min, the fibers were placed in the vacuum oven, at 60 °C, to remove the solvent mixture. After this stage, the fibers were placed in a 1 L vessel with deionized water and after about an hour, they could be used in the permeation process. Four types of membranes were obtained, symbolized according to Table 2.

### 2.3. Permeation Procedures

**Figure 4:**
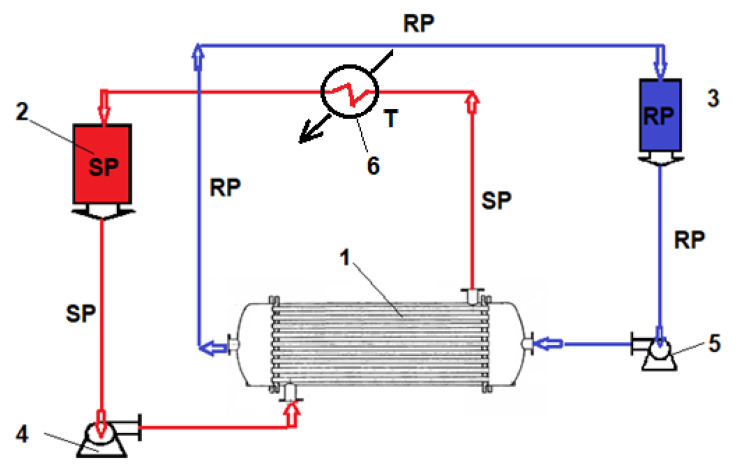
Depiction of the operational scheme with the pertraction module: SP – source phase, RS – receiving phase. **1**. Hollow fiber pertraction module; **2**. SP reservoirs; **3**. RP reservoirs; **4**. SP pump; **5**. RP pump; **6**. T thermostat.

In the source phase (SP) the synthetic solution of the considered chemical species (amino acids—Table 3), with a concentration of 0.150–0.250 mol/L, is introduced in the installation (Figure 4). The pH of the source phase is made with hydrochloric acid solution 0.01 mol/L, deionized water, or sodium hydroxide 0.1 mol/L. The receiving phase (RP) is formed in all cases from deionized water. The volume of the source phase is 10 L, and of the receiving phase is 1 L. The operating flows are the same for all experiments, for operational reasons already studied [69,70,71]. Thus, for the source phase the flow in the pertraction module is 5 L/min, and the flow of the receiving phase is 0.1 L/min. In order to determine the amino acids separation performances, a 1 mL of solution is taken periodically and analyzed for spectrophotometric analysis (CamSpec Spectrophotometer) [72,73,74]. For the receiving phase, the operation is performed through capillaries while for the source phase the operation is performed outside the capillaries (Figure 4).

The fluxes from the source phase [75] were determined against the measured permeate mass within a determined time range, applying the following equation:

(1) $$J=\frac{M}{S\cdot t}\;(\mathrm{mg}⁄(m^{2}\;s))\;\mathrm{or}\;((\mathrm{mol})⁄(m^{2}\;s))$$

where: *M—*permeate mass (g or mol), *S—*effective surface of the membrane (m^2^), *t—*time (s) necessary to collect the permeate volume.

The extraction efficiency (*EE*%) for the species of interest using the concentration of the solutions [76] was calculated as follows:

(2) $$EE\left(\%\right)=\frac{\left(c_{0}-c_{f}\right)}{c_{0}}\cdot 100$$

where: *c_f_*—final concentration of the solute (considered chemical species), *c_0_*—initial concentration of solute (considered chemical species).

The same extraction efficiency can also be computed based upon the absorbance of the solutions, as in:

(3) $$EE\left(\%\right)=\frac{\left(A_{0}-A_{s}\right)}{A_{0}}\;\cdot 100$$

where: *A*_0_—initial absorbance of sample solution, *A*_s_—current absorbance of the sample.

### 2.4. Equipment

The microscopy studies, scanning electron microscope (SEM) and high resolution SEM (HR SEM), were performed using a Hitachi S4500 system (Hitachi High-Technologies Europe GmbH, Mannheim, Germany). Thermal characterizations were performed using a Netzsch Thermal Analyzer (Netzsch—Gerätebau GmbH, Selb, Germany).

The thermal analysis TG-DSC for the cellulose samples (~20 mg) was performed with a Netzsch STA 449C Jupiter apparatus. The samples were placed in an open crucible made of alumina and heated with 10 K·min^−1^ from room temperature up to 900 °C, under the flow of 50 mL min^−1^ dried air. An empty alumina crucible was used as reference.

Spectroscopy Bruker Tensor 27 FTIR with a Diamond Attenuated Total Reflection—ATR (Bruker) was used to study the interactions between the chemicals used in the mem- branes developed. FTIR analysis was recorded in the range of 500 to 4000 cm^−1^. UV-VIS analysis was performed on a Spectrometer CamSpec M550 (Spectronic CamSpec Ltd., Leeds, UK). Other devices used were as follows: ultrasonic bath (Elmasonic S, Elma Schmidbauer GmbH, Singen, Germany), vacuum oven (VIOLA—Shimadzu, Bucharest, Romania).

## 3. Results and Discussion

The separation, concentration and purification of amino acids concerns researchers from various fields of activity: chemistry, biochemistry, engineering, medicine or the environment. Remarkable results were obtained in this field through membrane processes, but further research considers both the recovery of amino acids from poor sources and the creation of physical-chemically resistant biocompatible membranes—especially for sterilization or cleaning in order to reuse or maintain process performance.

Obviously, the concern of cellulose derivatives usage for amino acid recovery through membrane processes it is understandable. These derivatives are compatible with the biological environment and biological chemical species, in our case amino acids and their bio-resources. Likewise, the cellulose derivatives have a natural, specific interaction with amino acids, and the chemical modifications of this interaction are very important. The experimental study addressed three amino acids, two of which are essential amino acids. The three amino acids have different molar weights and solubilities, but the side chain, R, is hydrophobic of three types: alkyl (alanine), aromatic (phenylalanine) and sulfur (methionine). The hydrophobic radical of amino acids can interact with the cellulosic membrane material. For this reason the four chosen derivatives have substituents with etheric groups (HEC, MHEC), ester (CA) or carboxylic groups (NaCMC).

### 3.1. Membranes and Material Membranes Characterization

#### 3.1.1. Thermal Characteristics

**Figure 5:**
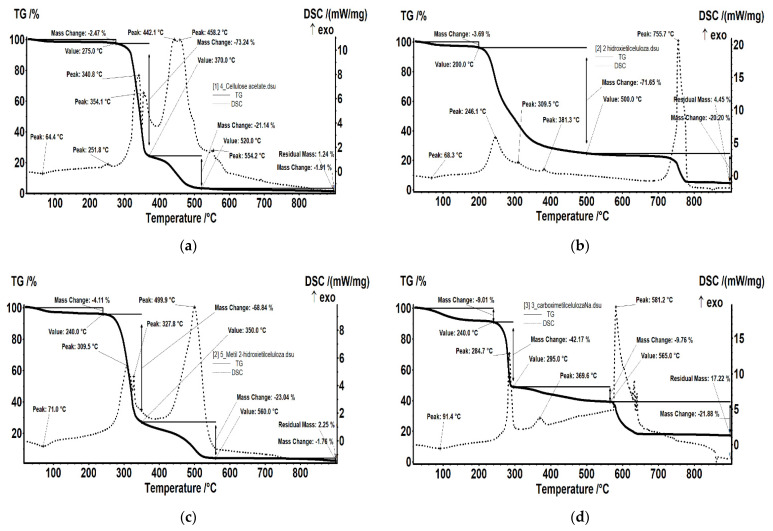
Thermal diagrams for: (**a**) cellulose acetate (CA); (**b**) 2–hydroxyethyl cellulose (HEC); (**c**) methyl 2–hydroxyethyl–cellulose (MHEC); (**d**) sodium carboxymethyl–cellulose (NaCMC).

**Figure 6:**
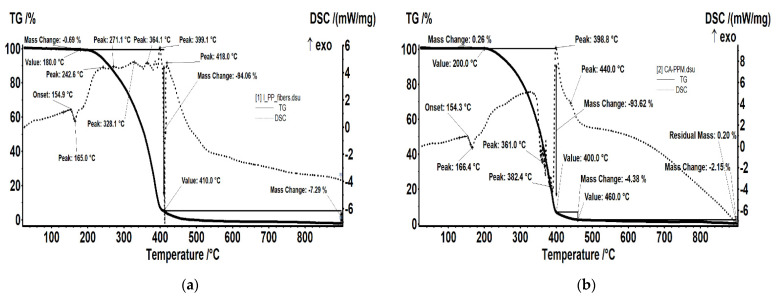

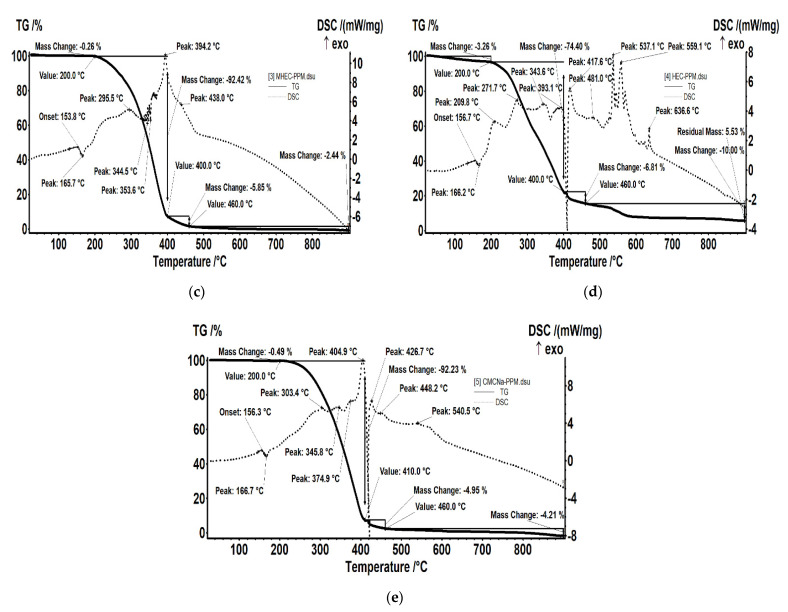
Thermal diagrams for hollow fiber membranes: (**a**) PPM; (**b**) CA–PPM; (**c**) MHEC–PPM; (**d**) HEC–PPM; (**e**) NaCMC–PPM.

Among the physical characteristics of the membranes and membrane materials proposed for study the mechanical ones are provided by the support matrix of polypropylene hollow fiber. But in the practice of membrane processes, the working temperature as well as the temperature of regeneration-washing or of sterilization is very important. Hence, the need to determine the thermal behavior of both membrane materials and impregnated membranes obtained (Figure 5 and Figure 6, Table 4 and Table 5 as well as the Supplementary Figures S1 and S2).

The cellulose acetate test (Figure 5a) is thermally stable up to 275 °C. The test loses 2.47% of its initial mass, most likely due to the water (moisture) absorbed, the process being accompanied by a weak endothermic effect at 64.4 °C. A weak exothermic effect is also observed at 251.8 °C, corresponding to a process of partial oxidation of the organic molecule. Degradative oxidation starts at 275 °C, the test loosing 73.24% up to 370 °C. The process is accompanied by two separate exothermic peaks, at 340.8 and 354.1 °C, corresponding to the degradation of the acetate and cellulose group. The carbon mass remaining after initial degradation is eliminate in the range 370–520 °C, through oxidation, the process being accompanied by an exothermic intense, wide effect, with two maxima at 442.1 and 458.2 °C.

The 2-hydroxyethyl cellulose test (Figure 5b) loses 3.69% of its initial mass in the range RT-200 °C, probably water absorbed by the hydroxyethyl cellulose powder. The process is accompanied by a weak endothermic effect, at 68.3 °C. After 200 °C the main process of oxidative degradation takes place, the mass loose continuing slowly until 500 °C. During this interval, 71.65% of the initial mass is lost. The process is accompanied by several exothermic effects with maxima at 246.1, 309.5 and 381.3 °C.

The methyl 2-hydroxyethyl cellulose test (Figure 5c) looses 4.11% of its initial mass, most likely the absorbed water, the process being accompanied by a weak endothermic effect, with a minimum at 71 °C. The degradative oxidation process took place in the interval 240–350 °C, the test loosing 68.84%. The process is accompanied by two separate, asymmetric, exothermic peaks at 309.5 and 327.8 °C, corresponding to the degradation of cellulose and to the lateral groups, respectively. The remaining carbon mass following the initial degradation is eliminated in the interval 350–560 °C, by oxidation, the process being accompanied by an exothermic, intense, wide, asymmetric effect with the maximum at 499.9 °C.

The sodium carboxymethyl-cellulose test (Figure 5d) looses 9.01% of its initial mass in the interval RT-240 °C, probably having a higher water content absorbed by the carboxymethyl-cellulose powder. The process is accompanied by a weak endothermic effect, at 91.4 °C. The main oxidative degradation takes place in the range 240–295 °C (42.17% of its initial mass is eliminated), the mass loss continuing slowly until 565 °C (another 9.76% is still eliminated). The processes are accompanied by exothermic effects with maxima at 284.7 °C and 369.6 °C. After 565 °C, there is a mass loss of 17.22% accompanied by a strong exothermic effect, with a maximum at 581.2 °C, most likely due to the decomposition of sodium carbonate and the reaction with the mass of the crucible material (Al_2_O_3_).

For impregnated membranes that have provided the best separation results (CA-PPM and MHEC-PPM), the thermal diagrams indicate behaviors close to the support fiber, but with specificities that must be taken into account, especially for operation, regeneration or thermal sterilization (Figure 6 and Table 5).

The sample PPM (Figure 6a) is relatively stable up to 180 °C, with 0.69% recorded mass loss. At the same time, a small endothermic effect is present on the DSC curve, with onset at 154.9 °C and a peak at 165.0 °C. This effect corresponds to the melting of the polypropylene. Between 180 and 410 °C the PPM fibers suffered an oxidative degradation, the recorded mass loss being 94.06%. The effects on DSC curve are a mix of endothermic (decomposing reactions) and exothermic (partial oxidation) effects, with the latest being more intense than former. In the 410–500 °C interval, the oxidation of the carbonaceous mass is achieved with a mass loss of 7.29%. The process is accompanied by a strong exothermic effect, asymmetric, with a maximum at 418.0 °C.

The sample CA-PPM (Figure 6b) is stable up to 200 °C, the recorded mass loss being 0.26%. At the same time, a small endothermic effect is present on the DSC curve, with onset at 154.3 °C and a peak at 166.4 °C. This effect corresponds to the melting of the polypropylene. Between 200 and 400 °C the sample presents a mass loss of 93.62%. The effects on DSC curve starts with an exothermic, broad one, with peaks at 240 and 327.5 °C, followed by a series of endothermic peaks. This indicates that first process is an oxidation of the polymeric fiber, followed by a series of decompositions. The carbonaceous mass obtained towards end decomposition starts to burn and a strong, sharp exothermic peak can be observed at 398.8 °C. The final oxidation process takes place quick up to 460 °C, with a recorded mass loss of 4.38%, and proceeds slowly after, with another 2.15% mass loss up to 900 °C.

The sample MHEC-PPM (Figure 6c) is stable up to 200 °C, the recorded mass loss being 0.26%. At the same time, a small endothermic effect is present on the DSC curve, with onset at 153.8 °C and a peak at 165.7 °C. This effect corresponds to the melting of the polypropylene. Between 200 and 400 °C the sample presents a mass loss of 92.42%. The first effect on DSC curve is exothermic, broad, asymmetric, with peaks at 235 and 295.5 °C, and indicates that some oxidation processes are responsible for the mass lost in the first part. There are multiple endothermic effects around 350 °C, which indicate the predominance of decomposition processes. The carbonaceous mass obtained by the end of the decomposition starts to burn and a strong, sharp exothermic peak can be observed at 394.2 °C. The final oxidation process takes place quickly up to 460 °C, with a recorded mass loss of 5.85%, and proceeds slowly after, with another 2.44% mass loss up to 900 °C.

The sample HEC-PPM (Figure 6d) is losing 3.26% of its initial mass up to 200 °C, indicating the presence of a small HEC quantity on fiber surface. In the same time, a small endothermic effect is present on the DSC curve, with onset at 156.7 °C and a peak at 166.2 °C. This effect corresponds to the melting of the polypropylene. Between 200 and 400 °C the sample exhibit a mass loss of 74.40%. The effects on DSC curve starts with a series of exothermic peaks at 209.8, 271.7, 343.6 and 393.1 °C and 327.5 °C indicating the presence of multiple degradative-oxidative processes. The carbonaceous mass obtained towards end decomposition starts to burn and a series of strong, sharp exothermic peaks can be observed at 417.6, 537.1 and 559.1 °C. In the final oxidation process, up to 600 °C, a mass loss of 10.00% is recorded.

The sample NaCMC-PPM (Figure 6e) is stable up to 200 °C, the recorded mass loss being 0.49%. A small endothermic effect is present on the DSC curve, with onset at 156.3 °C and a peak at 166.7 °C. This effect corresponds to the melting of the polypropylene fibers. Between 200 and 410 °C the sample presents a mass loss of 92.23%. The associated effects on DSC curve are exothermic, overlapped, with peaks at 303.4, 345.8, 374.9 and 404.9 °C, indicating the occurring of oxidation processes in this interval. The carbonaceous mass obtained starts to burn after 410 °C, when some strong, sharp exothermic peaks can be observed at 426.7 and 448.2 °C. In the final oxidation process the sample is losing 4.21% up to 900 °C.

#### 3.1.2. Chemical Structure and Composition

**Figure 7:**
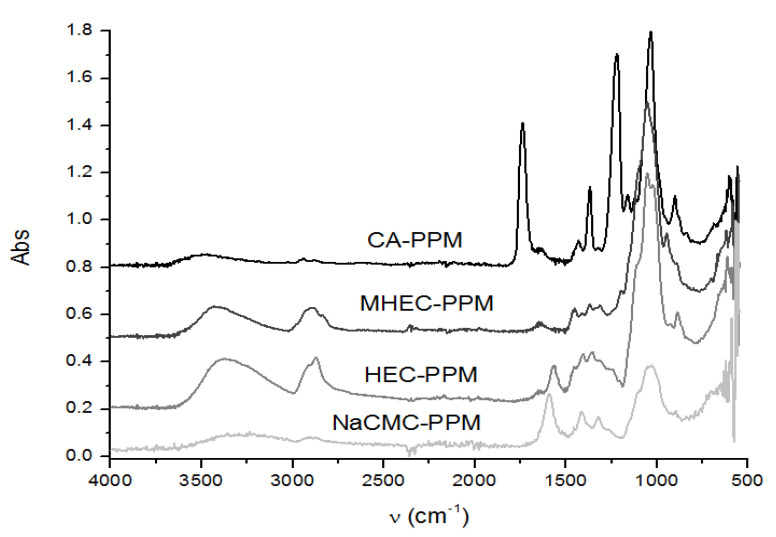
The Fourier transform infrared spectroscopy (FT–IR) spectrum of the impregnated membranes.

The chemical structure of the chosen cellulosic derivatives was highlighted through Fourier transform infrared spectroscopy (FT-IR), in which the functional groups that can interact specifically with the amino acids considered are observed (Figure 7 and Figure S3 and Table 6) Their characterization was obtained directly onto the solid samples, using the Bruker Tensor 27 with ATR diamond for the materials in this study.

The chemical structure of the four cellulosic derivatives which impregnate the membranes indicates possibilities of interaction with the amino acids or their ionic forms, both by hydrogen or dipole-dipole bonds (given by the carboxyl or amino groups) and by hydrophobic interactions of hydrocarbon chains. This statement is strengthened by the character of the radical R (Figure 1a) of the considered amino acids: aromatic hydrophobic (phenylalanine) or aliphatic hydrophobic (alanine and methionine).

Of course, depending on the pH of operation in the separation with the considered membranes, the complexity of the amino acid—cellulose derivative interaction must be taken into account. The superficial composition determined through energy dispersive spectroscopy analysis (EDAX) of impregnated membranes Cell-D-PPM compared with the PPM support membrane shows the appearance in different concentrations of oxygen atoms on the surface (Table 7 and Supplementary Figure S4).

This concentration of the oxygen atoms on the surface of the membrane will be responsible for the attraction of the amino acid, but the migration inside the impregnated membrane depends on the overall composition of the cellulose derivative used for impregnation.

#### 3.1.3. Membrane Morphology

The surface morphology of the considered samples was analyzed using a scanning electron microscope (SEM), high-resolution scanning electron microscope (HR-SEM). All samples were properly dried prior to the microscopy analysis and were sufficiently coated with a sputtered gold layer of 400 Å.

The results of the image analysis are presented in Figure 8, Figure 9, Figure 10 and Figure 11 (see Supplementary Figures S5–S8).

**Figure 8:**
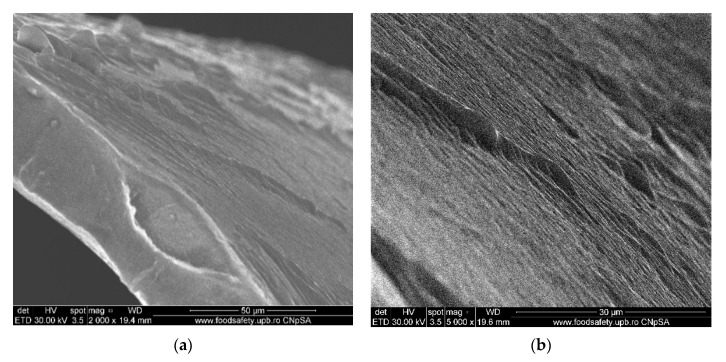
Morphology CA–PPM membranes: (**a**) membrane cross–section; (**b**) membrane surface.

**Figure 9:**
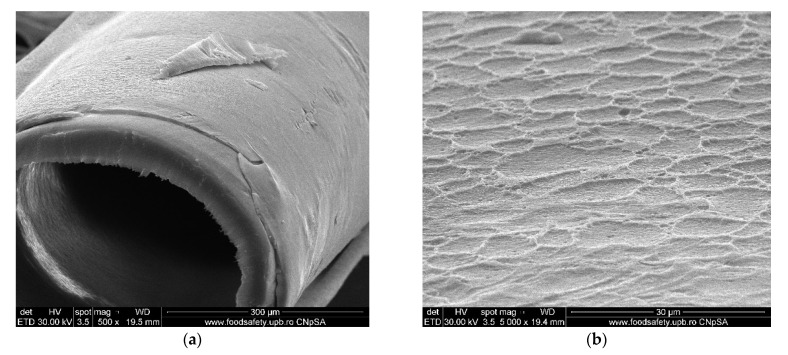
Morphology HEC–PPM membranes: (**a**) membrane cross–section; (**b**) membrane surface.

**Figure 10:**
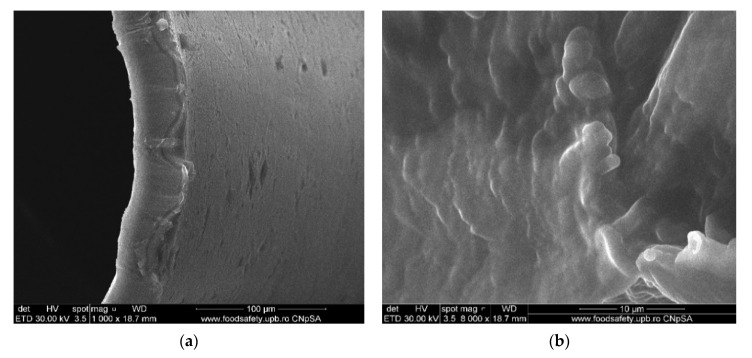
Morphology MHEC–PPM membrane: (**a**) membrane cross–section; (**b**) membrane surface.

**Figure 11:**
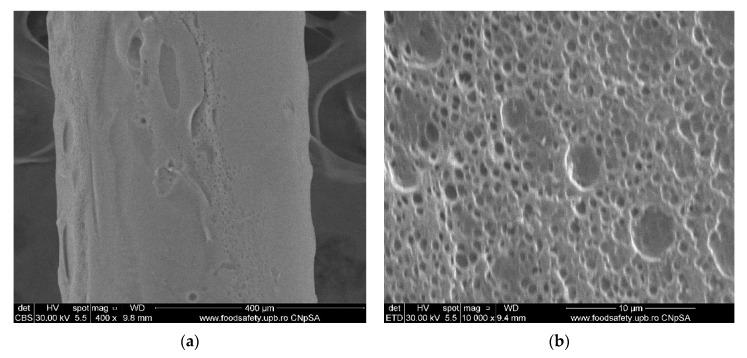
Morphology NaCMC–PPM membranes: (**a**) view; (**b**) membrane surface.

**Figure 12:**
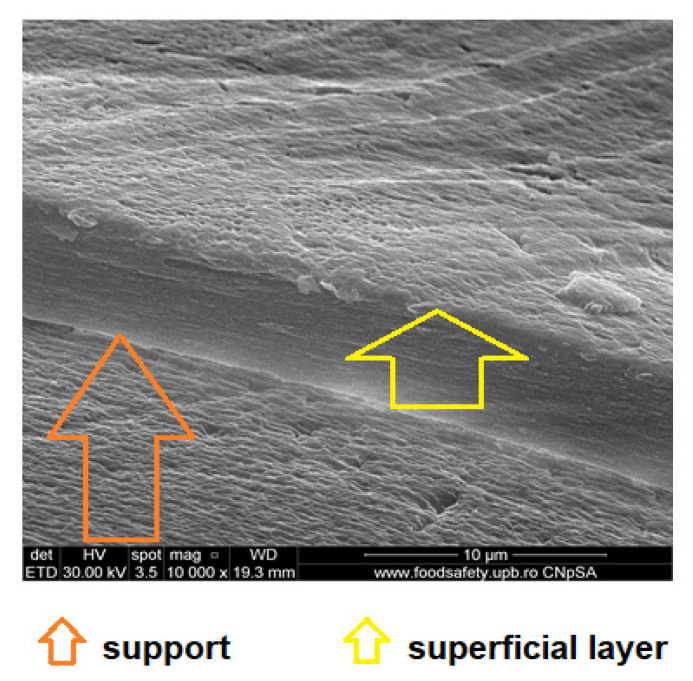
Detail of the superficial layer from the cellulose derivatives on the polypropylene hollow fiber.

**Figure 13:**
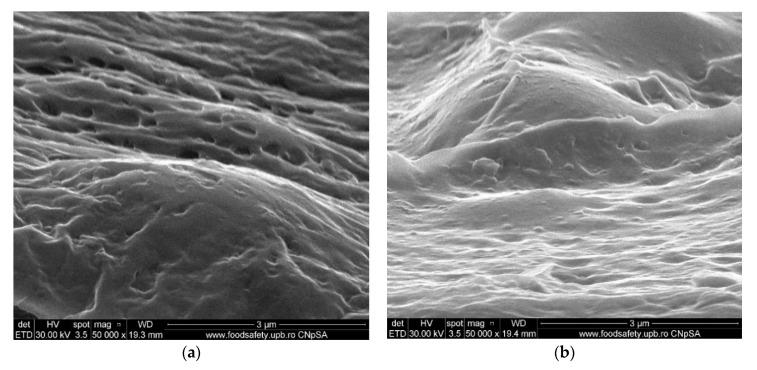
Detail of the superficial layer from the cellulose derivatives: (**a**) cellulose acetate and (**b**) 2–hydroxyethyl cellulose.

From the images obtained through scanning electron microscopy (SEM) and high resolution scanning electron microscopy (HR-SEM) some common features of the obtained impregnated membranes can be highlighted, but also several specificities, as follows:

- Impregnation of the membranes takes place superficially and adherently, without the cellulosic derivative reaching the inside of propylene hollow fiber membrane (Figure 8a,Figure 9a and Figure 10a).
- The layer of cellulosic derivative from the surface of the membranes has about 5 µm (Supplementary Figures S5–S8a,b) being highlighted in the detail depicted in Figure 12.

- The surface layer of cellulosic derivative has a microstructure specific to nanofiltration membranes (Figure 8b, Figure 9b, Figure 10b and Figure 11b), highlighted in two significant details in Figure 13.

### 3.2. Performance of the Amino Acids Removal Process

The separation of amino acids is very important, and the use of membranes based on functionalized biopolymers involves the careful study of working parameters and especially of operational ones, such as pH and temperature. That is why the main operating parameters and their influence on the evolution of the target chemistry species separation are presented.

**Figure 14:**
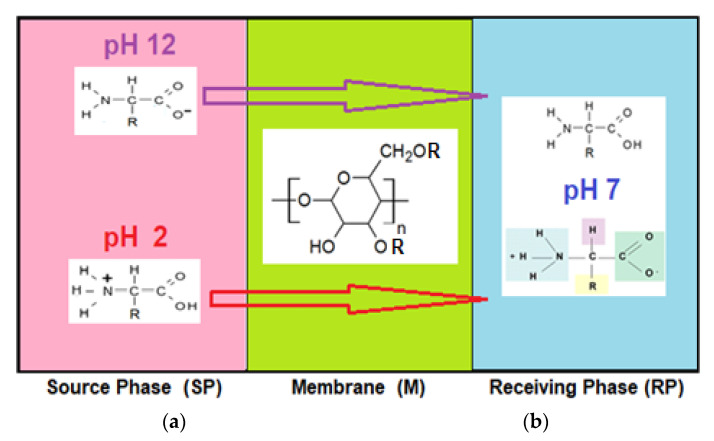
The transport mechanism of the amino acids at: (**a**) pH = 2 and pH = 12 for the source phase (SP); and (**b**) pH = 7 (pure water) in the receiving phase (RP).

The pH of the feeding solution was equal to 2 and 12, respectively, to have the certainty of the formation of the carboxyl anion, in the first case and of the ammonium cation, in the second case (Figure 1b and Table 3). The receiving phase consists of pure water (pH = 7) so that when re-extracted from the membrane the amino acid reaches the shape specific to the isoelectric point (Figure 14). The working temperature was 25 °C in the whole membrane system.

#### 3.2.1. The Effect of the Composition of the Impregnated Membrane on the Recovery of Amino Acids

**Figure 15:**
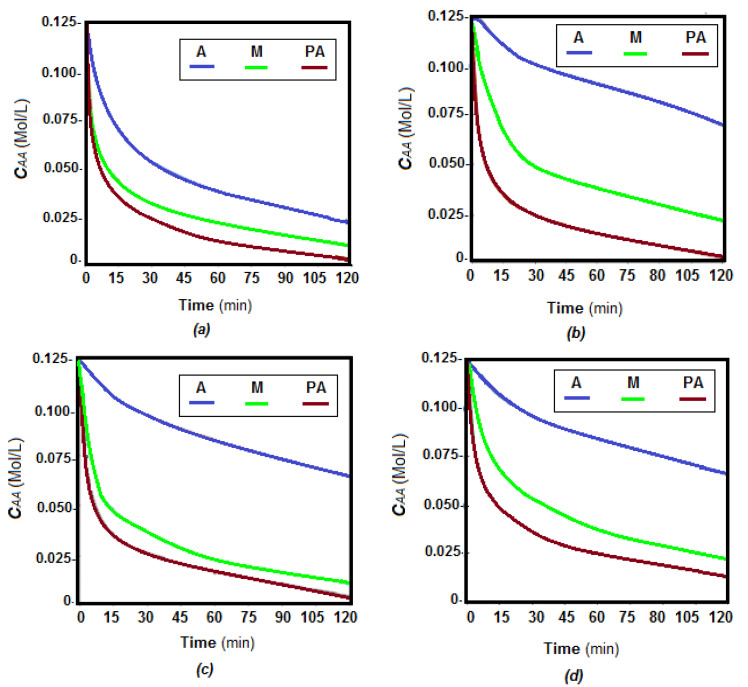
Variation of source phase amino acid concentration depending on the operating time at pH_SP_ = 12 and pH_RP_ = 7: (**a**) CA–PPM; (**b**) HEC–PPM; (**c**) MHEC–PPM; (**d**) NaCMC–PPM. **A** = alanine (blue); **M** = methionine (green) and **PA** = phenylalanine (brown).

**Figure 16:**
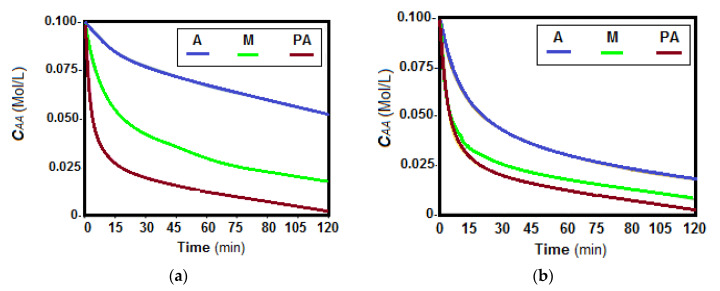

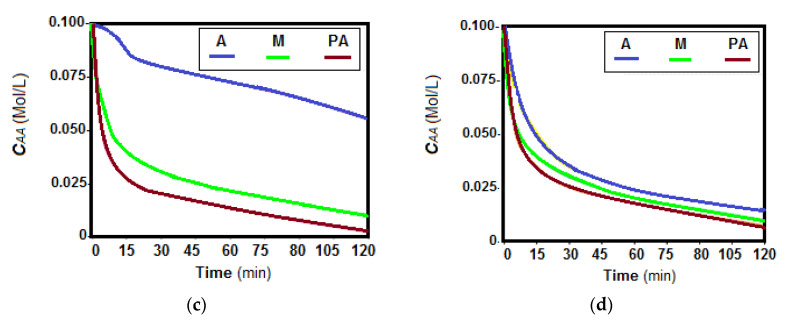
Variation of source phase amino acid concentration depending on the operating time at pH_SP_ = 2 and pH_RP_ = 7: (**a**) CA–PPM; (**b**) HEC–PPM; (**c**) MHEC–PPM; and (**d**) NaCMC–PPM. **A** = alanine (blue); **M** = methionine (green), and **PA** = phenylalanine (brown).

The first pertraction tests of the chosen amino acids, alanine, phenylalanine and methionine, considered the study of the evolution of amino acids in source phase, in a determined interval of 120 min (Figure 15 and Figure 16).

The concentration of the three amino acids decreases rapidly in the first 60 min of operation, after which the decrease is slower, or a level is created (Figure 15 and Figure 16). The lowest decrease rate is in all cases for alanine, followed by methionine, and phenylalanine.

However, some peculiarities stood out:

- The difference in transfer rate was large between alanine and the other two amino acids, especially for HEC-PPM, MHEC-PPM and NaCMC-PPM membranes, at pH = 12 and for CA-PPM and MHEC-PPM membranes, at pH = 2,
- The transfer rates differed less in the case of CA-PPM at pH = 12 and for HEC-PPM and NaCMC at pH = 2.

Considering the amino acid flows (Table 8), depending on the nature of cellulose derivative that forms the impregnated membrane and the surface composition (Table 7), it can be seen that there were significant differences in the flow of the three amino acids.

The results obtained show that phenylalanine has the highest fluxes through all four types of membranes, followed by methionine and alanine. pH does not change this order but suggests the possibility of selective separation of the three amino acids, more obvious being the separation of alanine from phenylalanine.

Of the four kinds of membrane, the most suitable for recuperative separation of the considered amino acids are those based on cellulose acetate and methyl 2-hydroxyethyl-cellulose.

#### 3.2.2. The Effect of pH and Solubility of Amino Acids on the Flow through Impregnated Membranes

**Figure 17:**
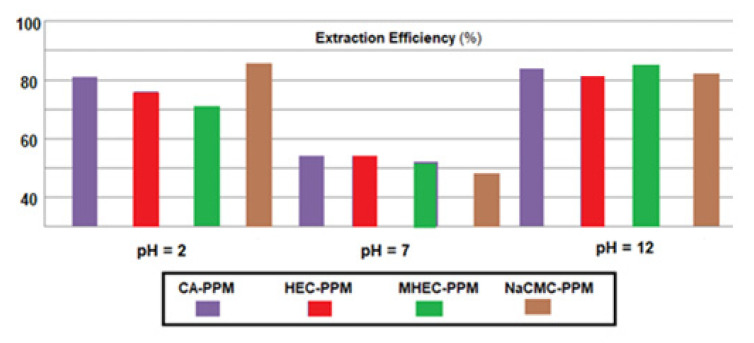
The phenylalanine extraction efficiency EE (%) variation versus the pH of the source phase (SP) for the prepared membranes.

The flow performances were also found in the efficiency of phenylalanine extraction, chosen for the highest transfer rates found in all previous experiments for all four types of membrane (Figure 17). Depending on the pH of the source phase, the following succession was obtained:

(4) $$EE_{pH\;12}=EE_{pH\;2}>EE_{pH\;7}$$

**Figure 18:**
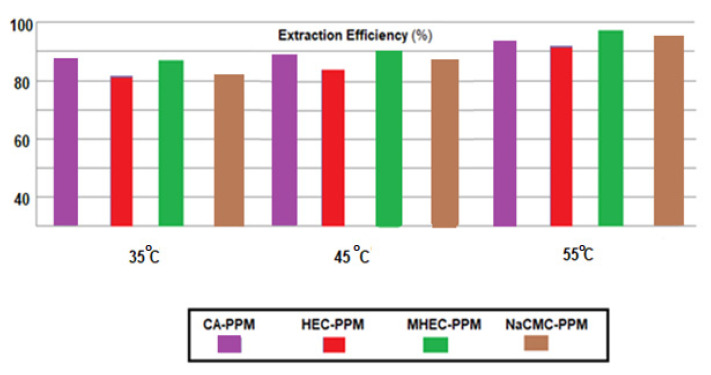
The phenylalanine extraction efficiency EE (%) variation versus the temperature of source phase (SP) for the prepared membranes.

Determination of the efficiency of extraction of the amino acids from source solutions of pH = 12, at temperatures of 35, 45 and 55 °C, shows that the separation efficiency grows with increasing temperature for all four types of membranes (Figure 18). A more pronounced effect can be seen in the case of HEC-PPM and NaCMC-PPM membranes, which could correlate with higher hydrophilicity of membranes based on the two cellulosic derivatives. However, the usage of these impregnation derivatives is limited by the solubility of the polymeric layer in the source solution, which would lead over time to significant losses and drastic decreases in membrane thickness, known in the literature of the membranes as ‘membrane washing’ [77].

The transfer rate of alanine is lower in all cases, remaining in high concentration in the source phase. This observation suggests the possibility of separating the three chosen amino acids by the appropriate choice of the cellulosic derivative. Thus, phenylalanine and methionine can be recovered from the receiving phase and alanine is concentrated in the source phase.

One explanation that would answer to the results presented in Figure 13, Figure 14 and Figure 15a would be that the solubility of alanine is much higher than that of the other two amino acids (Table 3), which would lead to an important retention of it in the source phase.

The argument presented is even more valid as the transfer of phenylalanine is in all cases higher than that of methionine. Thus, it could be generalized that that the transfer flow (***J***) of amino acids through the studied membranes grows in this order:

(5) $$J_{phenylalanine}>J_{methionine}>J_{alanine}$$

which would thus be correlated with the solubility in pure water (***So***) of the amino acids:

(6) $$So_{phenylalanine}<So_{methionine}<So_{alanine}$$

The results can be explained by the fact that in aqueous solution the amino acids participate in proton exchange equilibria which are controlled by pH:

HOOC-CHR-NH_2_ + H_3_O^+^ ↔ HOOC-CHR-NH_3_^+^ + HOH (7)

HOOC-CHR-NH_2_ + HOH ↔^−^OOC-CHR-NH_2_ + H_3_O^+^ (8)

Based on these balances, the acidity constants are defined:

(9) $$K_{a_{1}}=\frac{\left[HOOC-CHR-NH_{2}\right]\left[H_{3}O^{+}\right]}{\left[HOOC-CHR-NH_{3}^{+}\right]}$$

(10) $$K_{a_{2}}=\frac{\left[OOC-CHR-NH_{2}\;\right]\left[H_{3}O^{+}\;\right]}{\left[HOOC-CHR-NH_{2}\right]}$$

The degree of formation of these chemical species can be assessed with the following relationships:

(11) $$\alpha_{0}=\frac{1}{1+10^{pK_{a_{2}}-pH}+10^{pK_{a_{1}}+pK_{a_{2}}-2pH}}$$

(12) $$\alpha_{1}=\frac{1}{1+10^{pK_{a_{1}}-pH}+10^{pH-pK_{a_{2}}}}$$

(13) $$\alpha_{2}=\frac{1}{1+10^{pH-pK_{a_{1}}}+10^{2pH-pK_{a_{1}}-pK_{a_{2}}}}$$

In which α_0_, α_1_ and α_2_ represent, respectively, the degrees of formation of the species ^–^OOC-CHR-NH_2_, HOOC-CHR-NH_2_ and HOOC-CHR-NH_3_^+^, in the aminophenol solution.

The actual solubility (*S*) of the considered amino acid is a function of pH and the solubility in pure water (*S*_0_), which explains the behavior of the amino acid in the pertraction process:

(14) $$S=S_{0}\;\cdot f\left(\alpha \right)$$

**Figure 19:**
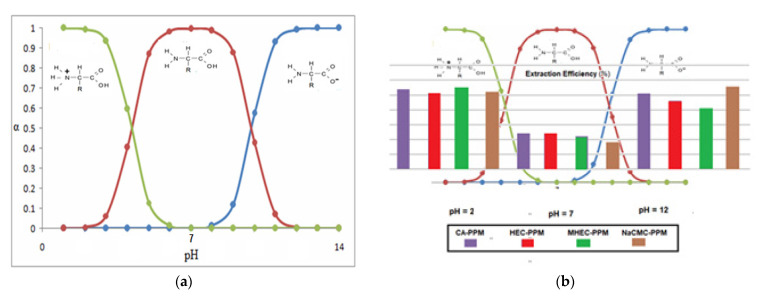
The speciation diagram of an amino acid, as a function on pH: (**a**) diagram; and (**b**) the diagrams superimposed over the extraction efficiency graph from Figure 17.

With these relations one can illustrate the speciation diagram for a generic amino acid with: *pK_a_*_1_ = 2.30 and *pK_a_*_2_ = 9.20 (Figure 19a):

The results obtained in this paper are well illustrated by overlapping the diagram in Figure 17 with that of the chemical speciation of amino acids as function of pH (Figure 19a). Thus, it can be observed (Figure 19b) that if the pH of the source phase varies from 2 to 7 and then to 12, while keeping constantly the pH of the receiving phase at 7, then the extraction efficiency follows a winding curve that is specific to the existence of the three forms of the amino acid.

Based on the analysis of the speciation diagram, the pH conditions for the formation of the different chemical species of the studied amino acids can be anticipated, so as to obtain the optimal results in separation.

## 4. Conclusions

Amino acids are substances whose chemical and biochemical impact are quite remarkable. A special place in the study of the amino acids is occupied by their recovery from various sources, through a multitude of separation methods and techniques in which the membranes occupy a special place.

The present paper addressed the recuperative separation of three amino acids (alanine, phenylalanine and methionine) from synthetic solutions, using membranes from cellulose derivatives (cellulose acetate, 2-hydroxyethyl-cellulose and methyl 2-hydroxylethyl-cellulose or sodium carboxymethyl-cellulose) in the polypropylene hollow fiber matrix.

In order to determine the basic characteristics, the separation performances of the considered amino acids (retention, flow and selectivity) and the morphological and structural ones, the membranes and membrane materials were analyzed by specific techniques: scanning electron microscopy (SEM), high resolution SEM (HR-SEM), Fourier transform infrared spectroscopy (FT-IR), energy dispersive spectroscopy (EDS) and thermal-gravimetric analyzer (TGA).

The support offered by polypropylene hollow fibers confers physical-chemical resistance, and cellulosic derivatives used for impregnation contribute to the separation performances of prepared impregnated membranes.

The results of the separation were influenced by the pH of the source phase and the solubility of the amino acids considered, the best results as flow and extraction efficiency being obtained when the source phase had a pronounced acidic or basic pH and the receiving phase had a neutral pH (pure water).

Among the amino acids, the highest transmembrane fluxes and extraction efficiency are offered by phenylalanine, then methionine and finally alanine. This succession is closely correlated with the solubility of the amino acids studied in water, a high solubility in water leading to a low transfer rate and separation efficiency.

By choosing the right cellulose derivative, phenylalanine and methionine can be recovered from the receiving phase, and alanine is concentrated in the source phase.

The considered cellulosic derivatives (cellulose acetate, 2-hydroxyethyl-cellulose, methyl 2-hydroxyethyl-cellulose or sodium carboxymethyl-cellulose) have a similar behavior in the separation process, but following the experiments, cellulose acetate and methyl 2-hydroxyethyl-cellulose are recommended for the realization of impregnated membranes. Although when increasing the operating temperature from 25 to 55 °C the performance of membranes impregnated with 2-hydroxyethyl-cellulose and sodium carboxymethyl-cellulose increased, still the use of these membranes at high temperature raised the problem of degradation by the solubilization-washing phenomenon.

## Figures and Tables

**Table 1:** Membrane amino acids separation techniques and methods.

Methods or Techniques	Gradient	Refs.
Dialysis (D), Hemodialysis (HD)	Δc	[20,21,22]
Ion-exchange membranes (IEM)	Δc, ΔE	[23,24,25]
Electro dialysis (ED)	ΔE	[26,27]
Electro-ultrafiltration (EUF)	ΔE, Δp	[28,29,30,31,32,33]
Ultrafiltration (UF)	Δp	[34,35,36,37]
Nanofiltration (NF)	Δp	[38,39,40,41,42,43]
Molecularly imprinted polymeric membranes (MIPM)	Δp	[44]
Liquid membranes (LM)	Δc	[45,46,47]
Other processes (Adsorption, Polyelectrolyte nanoparticles, Composite membranes, Combined processes, Molecular recognition)	Δc, ΔE, Δp	[48,49,50,51,52,53,54,55,56,57,58,59]

**Table 2:** The characteristics of cellulosic derivatives under test and the membranes obtained.

Cellulose Derivatives (Cell-D)	Chemical Formula	Molar Weight	Membrane Symbol
cellulose acetate (CA)	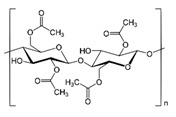	50,000	CA-PPM
2-hydroxy-ethylcellulose (HEC)	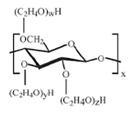	380,000	HEC-PPM
methyl 2-hydroxyethyl-cellulose (MHEC)	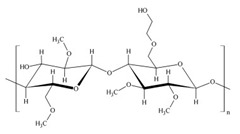	*)	CHEC-PPM
sodium carboxymethyl-cellulose (NaCMC)	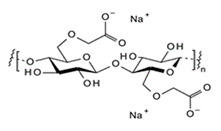	90,000	NaCMC-PPM

*) Viscosity (c = 2%, water, 20 °C) 15,000–20,500 cps.

**Table 3:** The characteristics of the tested amino acids (at 25 °C).

Amino Acid	Chemical Formula	Molar Weight (g/mol)	Solubility (g/L)	Isoelectric Point (Ip)	Acidity Constants (pKa)
Alanine	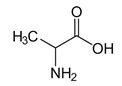	89.09	167.2	6.11	2.35 (carboxyl), 9.87 (amino)
Phenylalanine	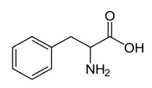	165.19	26.9	5.91	2.58 (carboxyl), 9.13 (amino)
Methionine or 2-amino-4-(methylthio) butanoic acid	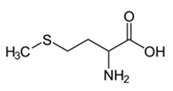	149.21	56.6	5.74	2.28 (carboxyl), 9.21 (amino)

**Table 4:** Main thermal characteristics of the used cellulose derivatives.

Sample	Water Content	Mass Loss 200–400 °C	Mass Loss 400–800 °C	Residual Mass
CA	2.47%	76.35%	20.31%	1.24%
HEC	3.71%	67.92%	23.17%	4.45%
MHEC	4.11%	73.63%	19.06%	2.25%
NaCMC	9.02%	47.84%	26.28%	17.22%

**Table 5:** Main thermal characteristics of the hollow fiber membranes.

Sample	PP Melting Onset (°C)	PP Melting Peak (°C)	Decomposition Start (°C)	T_10%_ (Temperature for 10% Mass Loss) (°C)
1_PP_fiber (support)	154.9	165.0	180	258
2 CA-PPM	154.3	166.4	200	274
3 MHEC-PPM	153.8	165.7	200	266
4 HEC-PPM	156.7	166.2	80	246
5 NaCMC-PPM	156.3	166.7	200	281

**Table 6:** The main structural characteristics identified through Fourier transform infrared spectroscopy (FT–IR).

Cellulose Derivatives	Wave Numbers of the Interesting Groups for the Considered Interactions (cm^−1^)					
	ν O-C- δ O-C-O	δ-CH_2_ δ O-H	δ-CH_2_	ν C=O	ν C-H	ν O-H
CA	1034	1371	1410	1743	2960 s	3499
HEC	1057	1352	1412	-	2987	3374
MHEC	1055	1371	1420	-	2896	3422
NaCMC	1034	1319	1418	-	2940	3280

ν = vibration; δ = deformation.

**Table 7:** The surface composition of the prepared impregnated membranes.

Membranes/ Composition	PPM		CA-PPM		HEC-PPM		MHEC-PPM		NaCMC-PPM	
	Weight%	Atomic%	Weight%	Atomic%	Weight%	Atomic%	Weight%	Atomic%	Weight%	Atomic%
C K	93.49	95.03	68.90	77.87	88.26	90.92	53.74	60.74	87.86	90.79
O K	6.51	4.97	31.10	28.23	11.74	9.08	46.26	39.26	11.27	8.74
Na K	-	-	-	-	-	-	-	-	0.88	0.47

**Table 8:** The fluxes of the cellulose derivatives membranes at pH = 2 and pH = 12 of the source phase (**A** = alanine; **M** = methionine, and **PA** = phenylalanine).

pH Source Phase	Membrane Flux (µMol/m^2^·s)											
	CA-PPM			HEC-PPM			MHEC-PPM			NaCMC-PPM		
	A	M	PA	A	M	PA	A	M	PA	A	M	PA
2	83.3	186.1	241.6	180.5	222.5	244.5	69.4	208.5	227.8	194.4	229.0	230.8
12	236.1	277.0	300.4	83.2	235.6	305.4	111.4	280.5	305.5	97.2	236.1	278.0

## Data Availability

Not applicable.

## Supplementary Materials

The following are available online at https://www.mdpi.com/article/10.3390/membranes11060429/s1, Figure S1 and S2: The superposed thermal diagrams; Figure S3: FTIR spectra of the obtained membranes; Figure S4: The compositional surface (EDAX) of the support membrane and two impregnated membrane; Figures S5-S8: The results of the image analysis (SEM).
