# Supplementary material for: Recuperative Amino Acids Separation through Cellulose Derivative Membranes with Microporous Polypropylene Fiber Matrix
Source: Membranes (Basel). 2021 Jun 5;11(6):429. doi: 10.3390/membranes11060429 (PMC8228197; doi:10.3390/membranes11060429)
Supplement: Supplementary file 1 [file membranes-11-00429-s001.zip › membranes-1223153-supplementary.pdf]

membranes

Supplementary Material

Recuperative Amino Acids Separation through Cellulose Derivative Membranes with Microporous Polypropylene Fiber Matrix

Aurelia Cristina Nechifor [1], Andreia Pîrțac [1], Paul Constantin Albu [2], Alexandra Raluca Grosu [1,*], Florina Dumitru [3], Ioana Alina Dimulescu (Nica) [1], Ovidiu Oprea [3], Dumitru Pașcu [1], Gheorghe Nechifor [1] and Simona Gabriela Bungău [4]

[1] Analytical Chemistry and Environmental Engineering Department, University Politehnica of Bucharest, 1-7 Polizu St., 011061, Bucharest, Romania; aureliacristinanechifor@gmail.com (A.C.N.); andreia.pascu@yahoo.ro (A.P.); oanaalinadimulescu@yahoo.com (I.A.D.); dd.pascu@yahoo.com (D.P.); ghnechifor@gmail.com (G.N.)
[2] IFIN Horia Hulubei, Radioisotopes and Radiation Metrology Department (DRMR), 30 Reactorului St., 023465 Măgurele, Romania; paulalbu@gmail.com
[3] Department of Inorganic Chemistry, Physical Chemistry and Electrochemistry, University Politehnica of Bucharest, 1-7 Polizu St., 011061 Bucharest, Romania; d_florina@yahoo.com (F.D.); ovidiu73@yahoo.com (O.O.)
[4] University of Oradea, Faculty of Medicine and Pharmacy, Universității St., no.1, 410087 Bihor, Oradea, Romania; simonabungau@gmail.com
* Correspondence: alexandra.raluca.miron@gmail.com

Abstract: The separation, concentration and transport of the amino acids through membranes have been continuously developed due to the multitude of interest amino acids of interest and the sources from which they must be recovered. At the same time, the types of membranes used in the separation of the amino acids are the most diverse: liquids, ion exchangers, inorganic, polymeric or composites. This paper addresses the recuperative separation of three amino acids (alanine, phenylalanine, and methionine) using membranes from cellulosic derivatives in polypropylene matrix. The microfiltration membranes (polypropylene hollow fibers) were impregnated with solutions of some cellulosic derivatives: cellulose acetate, 2-hydroxyethyl-cellulose, methyl 2-hydroxyethyl-celluloseand sodium carboxymethyl-cellulose. The obtained membranes were characterized in terms of the separation performance of the amino acids considered (retention, flux, and selectivity) and from a morphological and structural point of view: scanning electron microscopy (SEM), high resolution SEM (HR-SEM), Fourier transform infrared spectroscopy (FT-IR), energy dispersive spectroscopy (EDS) and thermal gravimetric analyzer (TGA). The results obtained show that phenylalanine has the highest fluxes through all four types of membranes, followed by methionine and alanine. Of the four kinds of membrane, the most suitable for recuperative separation of the considered amino acids are those based on cellulose acetate and methyl 2-hydroxyethyl-cellulose.

Keywords: amino acids; cellulose derivatives; polypropylene hollow fibers; impregnated membranes; amino acid separation; membrane processes.

Thermal analysis

The thermal analysis generated the results materialized in the superposed diagrams presented in Figure S1 and Figure S2, representing analysis results (TG and DSC) for cellulose derivatives, polypropylene hollow fibers, and impregnated polypropylene hollow fibers. The diagrams were recorded up to 900°C, with a heating speed of 10 °C·min^{-1}.

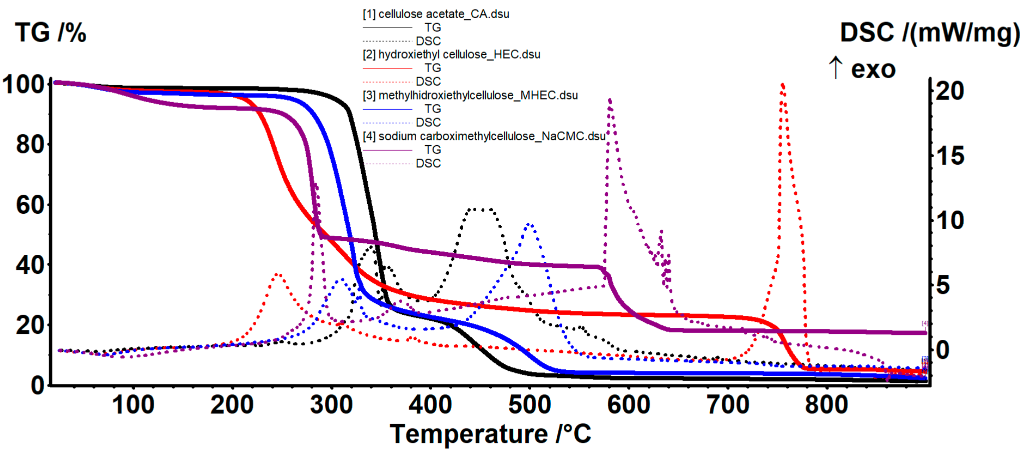

Figure S1. The superposed thermal diagrams for: (**1**) cellulose acetate (CA); (**2**) 2–hydroxyethyl cellulose (HEC); (**3**) methyl 2–hydroxyethyl–cellulose (MHEC); and (**4**) sodium carboxymethyl–cellulose (NaCMC).

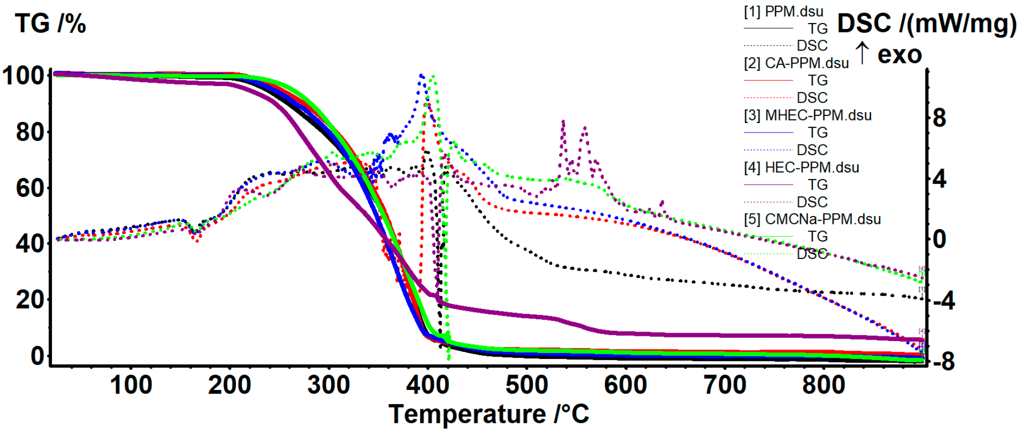

Figure S2. The superposed thermal diagrams for: (**1**) polypropylene hollow fibers (PPM); (**2**) CA-PPM impregnated polypropylene fibers; (**3**) MHEC–PPM impregnated polypropylene fibers; (**4**) HEC–PPM impregnated polypropylene fibers; and (**5**) NaCMC–PPM impregnated polypropylene fibers.

FTIR analysis

Figure S3 presents the FTIR spectra of the obtained membranes. Their characterization was obtained directly onto the solid samples, using the Bruker Tensor 27 with ATR diamond for the materials in this study.

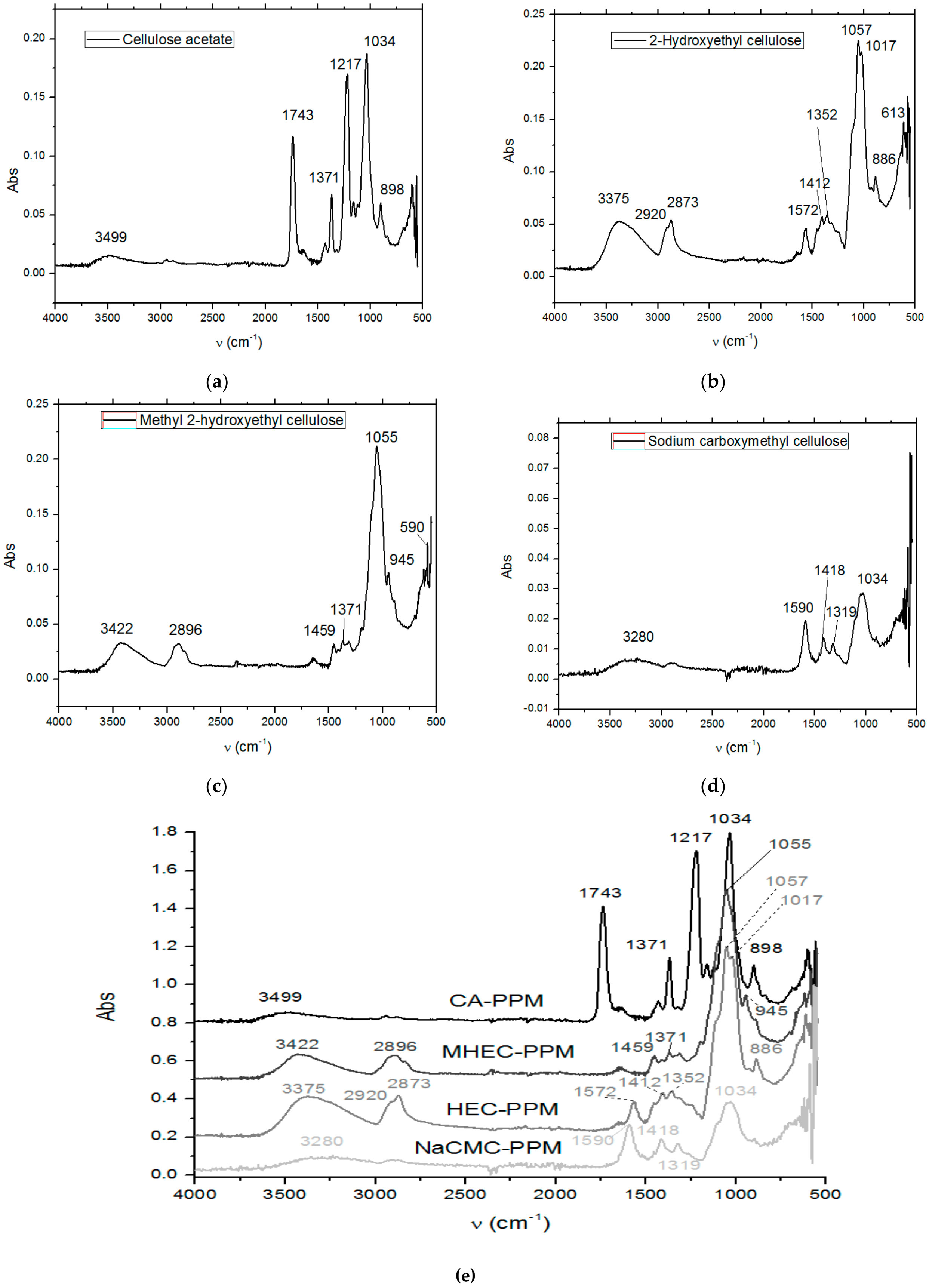

Figure S3. The Fourier transform infrared spectroscopy (FT–IR) spectrum of the impregnated membranes: (**a**) CA–PPM; (**b**) HEC–PPM; (**c**) MHEC–PPM; (**d**) NaCMC–PPM; **(e)** superposed FTIR spectra.

Surface analysis

The compositional surface (EDAX) of the support membrane and two impregnated membranes were illustrated in Figure S4.

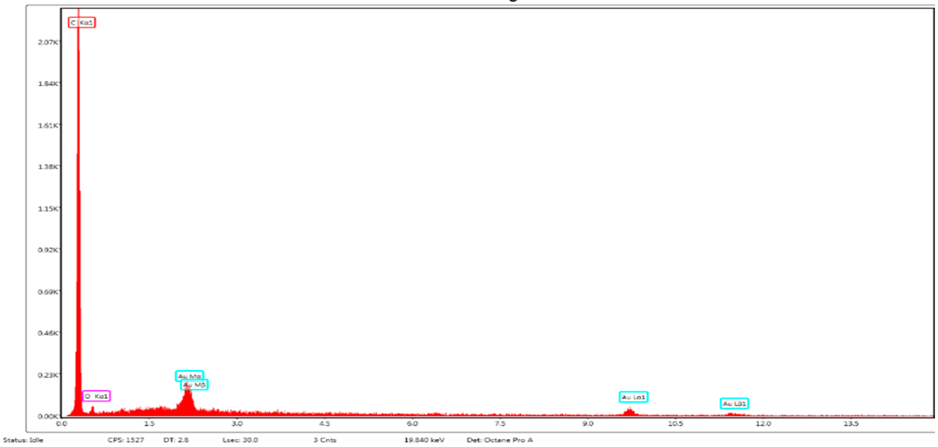

(**a**)

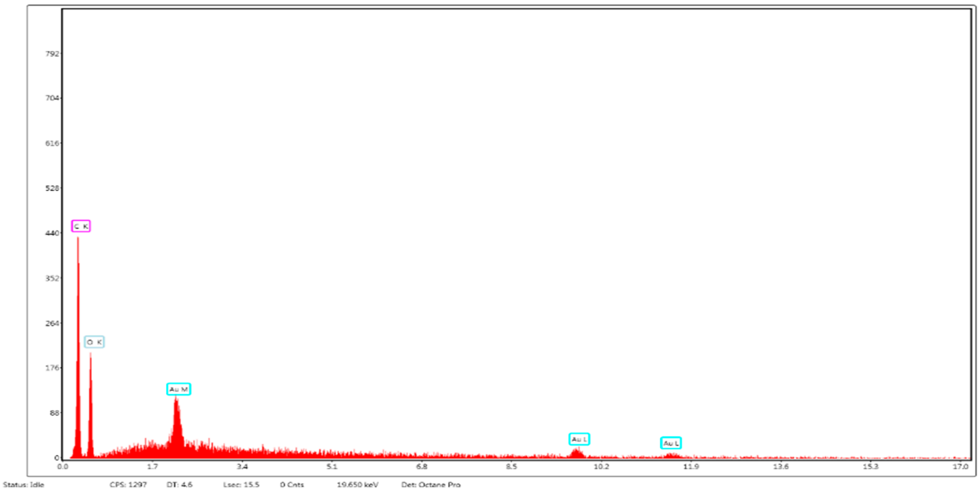

(**b**)

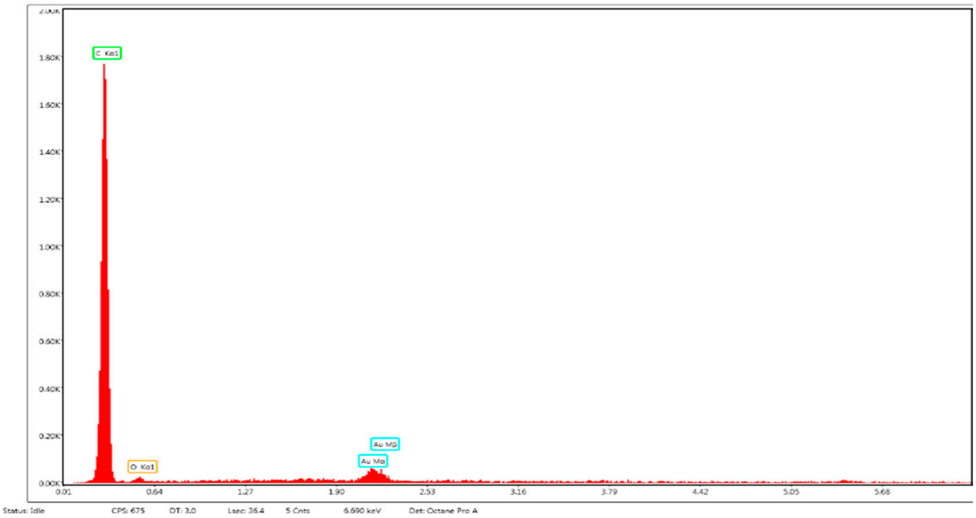

(**c**)

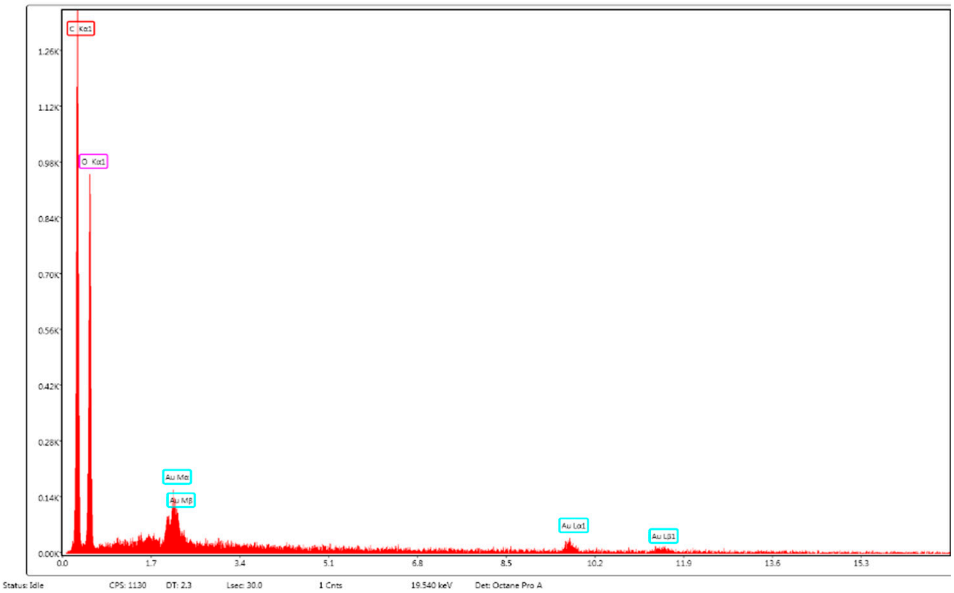

(d)

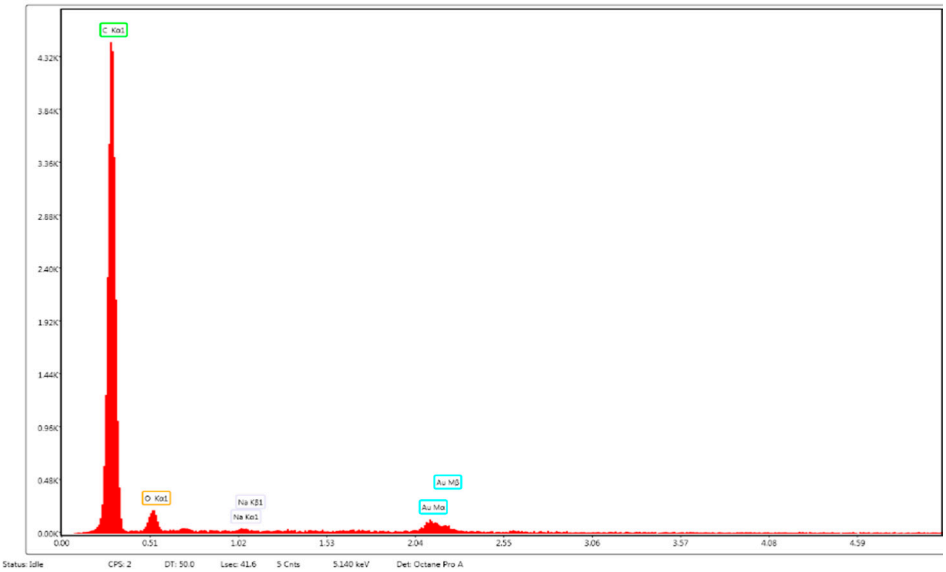

(e)

Figure S4. Energy dispersive X–ray analysis (EADX) on: (**a**) polypropylene hollow fiber membrane (PPM); (**b**) impregnated membranes (CA–PPM), **(c)** impregnated membranes (HEC–PPM), (**d**) impregnated membranes (MHEC–PPM), (**e**) impregnated membranes (NaCMC–PPM).

Scanning electron microscopy (SEM)

The results of the image analysis (SEM) are presented in Figures S5, S6, S7 and S8.

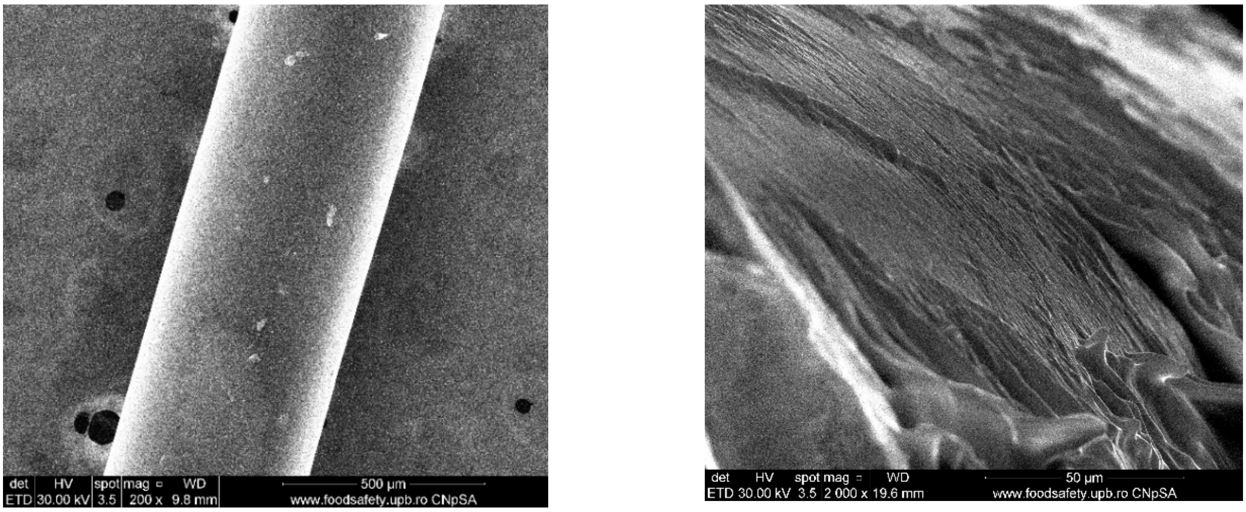

(**a**) (**b**)

Figure S5. Morphology CA–PPM membranes: (**a**) view; (**b**) membrane surface.

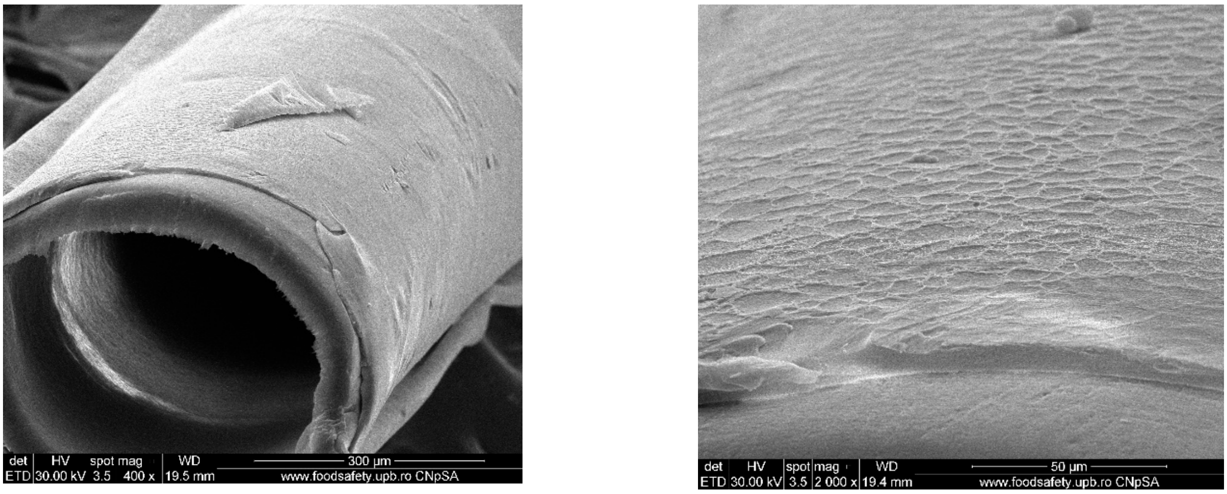

(**a**) (**b**)

Figure S6. Morphology HEC–PPM membranes: (**a**) view; (**b**) membrane surface.

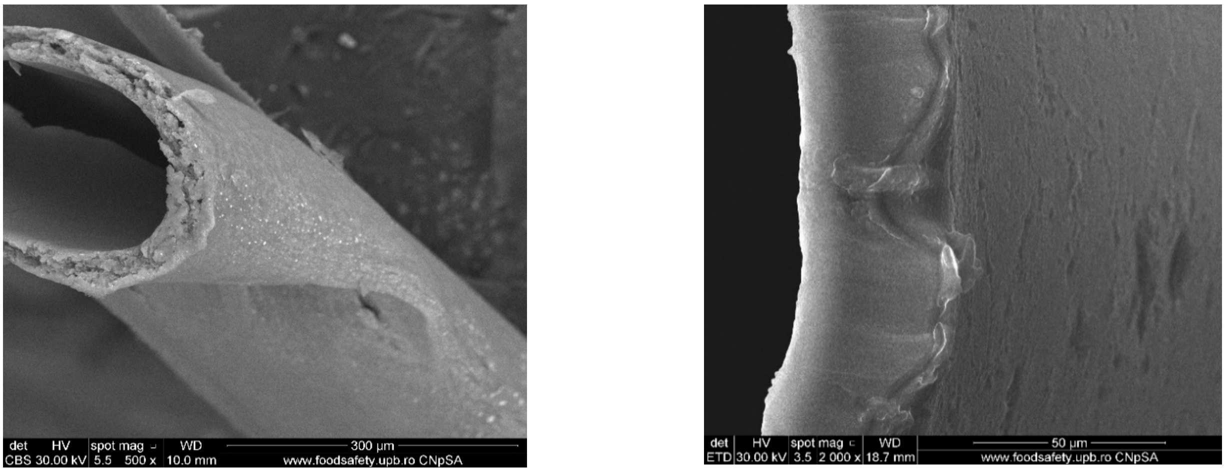

(**a**) (**b**)

Figure S7. Morphology MHEC–PPM membrane: (**a**) membrane cross–section; (**b**) membrane surface.

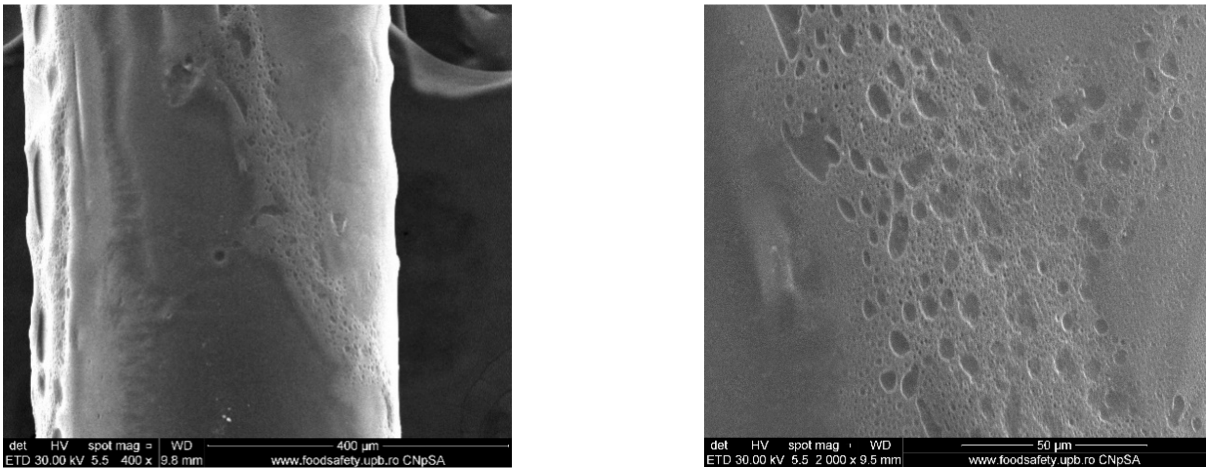

(**a**) (**b**)

Figure S8. Morphology NaCMC–PPM membranes: (**a**) view; (**b**) surface.
